# Ocular Clues in Musculoskeletal Disorders: A Narrative Review for Orthopaedic Specialists

**DOI:** 10.5435/JAAOSGlobal-D-25-00373

**Published:** 2026-09-08

**Authors:** Sara Alhomaidhi, Hamza M. Alrabai, Laila Alsabbagh, Abdullah Addar

**Affiliations:** From the College of Medicine (Alhomaidhi), King Saud University, Riyadh, Saudi Arabia; the Department of Orthopedics (Alrabai, Addar), College of Medicine, King Saud University, Riyadh, Saudi Arabia; and the Department of Orthopedic Surgery (Alsabbagh), King Abdullah bin Abdulaziz University Hospital, Riyadh, Saudi Arabia.

## Abstract

Musculoskeletal disorders (MSDs) are primarily characterized by joint, bone, and connective tissue involvement, but they can also present with ocular manifestations that carry diagnostic and clinical significance. These findings such as uveitis, blue sclera, lens subluxation, and scleritis may serve as early markers of systemic disease or indicate ongoing inflammation that affects orthopaedic decision making. Despite their relevance, ocular symptoms are frequently underrecognized in orthopaedic settings, potentially delaying comprehensive care.

This narrative review aims to explore the spectrum of MSDs with documented ocular involvement, examining the underlying pathophysiological mechanisms, clinical implications for orthopaedic specialists, and current recommendations for interdisciplinary management and ophthalmology referral. A literature search was conducted using PubMed, Scopus, and Google Scholar, including peer-reviewed studies published between 2016 and 2025. High-risk conditions, such as osteogenesis imperfecta, Marfan syndrome, rheumatoid arthritis, and juvenile idiopathic arthritis, were emphasized because of their ocular complexity and orthopaedic relevance.

Ophthalmologic evaluation in selected musculoskeletal populations can aid in early diagnosis, improve perioperative safety, and support holistic patient care. Incorporating structured identification of patients requiring ophthalmology referral into orthopaedic protocols, especially for syndromic, pediatric, and inflammatory conditions, represents a practical step toward more comprehensive musculoskeletal management.

Musculoskeletal disorders (MSDs) are a leading cause of disability worldwide, affecting millions of individuals with chronic pain, joint dysfunction, and skeletal abnormalities. Although primarily associated with the bones, joints, muscles, and connective tissues, many MSDs exhibit distinctive ocular manifestations that can serve as diagnostic markers, indicators of disease severity, or predictors of systemic complications. Despite these well-established associations, ocular findings are often overlooked in orthopaedic assessments, potentially delaying comprehensive diagnosis and treatment.

A wide range of MSDs including inflammatory, degenerative, metabolic, and genetic conditions demonstrate characteristic eye findings. Inflammatory diseases such as rheumatoid arthritis (RA), systemic lupus erythematosus (SLE), and ankylosing spondylitis (AS) are frequently associated with ocular conditions, including uveitis, keratoconjunctivitis sicca, and scleritis.^1^ Similarly, connective tissue disorders like Marfan syndrome and Ehlers-Danlos syndrome often present with lens subluxation, high myopia, and retinal detachment, which may precede musculoskeletal complications.^2^ Genetic conditions such as osteogenesis imperfecta (OI) exhibit blue sclera, a distinctive ocular feature that aids in the early recognition of bone fragility disorders.^3^ In addition, metabolic bone diseases like Paget disease have been linked to optic neuropathy, emphasizing the systemic nature of musculoskeletal disorders.^4^

From an orthopaedic perspective, recognizing ocular manifestations is crucial not only for early diagnosis but also for treatment planning and surgical decision making. For instance, uveitis in AS may indicate persistent systemic inflammation, which can increase fracture risk, delay spinal fusion surgery, and affect long-term mobility.^5^ Likewise, ocular signs in connective tissue disorders such as Marfan syndrome may alert orthopaedic surgeons to underlying aortic pathology, influencing surgical timing and perioperative management.^2^ Furthermore, chronic corticosteroid therapy for ocular inflammation in MSDs is a well-documented contributor to osteoporosis, which must be factored into fracture prevention and joint replacement planning.^6^

Despite these well-documented connections, ocular assessments remain underutilized in orthopaedic settings, and current screening protocols for musculoskeletal patients vary widely. Expanding orthopaedic guidelines to include routine eye evaluations could enhance preoperative risk stratification, optimize long-term musculoskeletal outcomes, and provide a more comprehensive approach to patient care.^1^

Although recent research has improved our understanding of MSD-related ocular manifestations, significant gaps remain in clinical practice. Many MSDs are systemic in nature, and their ocular findings may serve as crucial diagnostic markers that warrant further exploration. This narrative review examines the full spectrum of MSD-related ocular conditions, emphasizing their clinical relevance in orthopaedic practice, the need for interdisciplinary collaboration, and the importance of standardized screening protocols to improve patient outcomes.

## Methodology

This narrative review was conducted to explore the ocular manifestations associated with musculoskeletal disorders (MSDs), with a focus on their clinical implications for orthopaedic practice. The objective was to identify relevant conditions where eye findings aid diagnosis, indicate disease activity, or influence orthopaedic treatment and surgical planning.

### Search Strategy

A comprehensive literature search was performed using PubMed, Google Scholar, and Scopus. The search was conducted using combinations of keywords such as: “musculoskeletal disorders,” “ocular manifestations,” “uveitis,” “blue sclera,” “lens dislocation,” “connective tissue disorders,” “orthopedic implications,” and “inflammatory arthritis.”

### Inclusion Criteria

- Articles published in English between 2016 and 2025
- Peer-reviewed original research, systematic reviews, narrative reviews, and meta-analyses
- Studies addressing both musculoskeletal and ophthalmologic aspects
- Articles discussing clinical relevance to orthopaedic diagnosis, surgical care, or systemic management

### Exclusion Criteria

- Case reports and anecdotal literature without broader clinical implications
- Articles focused solely on ophthalmologic disease without musculoskeletal relevance
- Non–peer-reviewed sources, unpublished theses, and conference abstracts

### Selection and Data Extraction

All articles retrieved from initial database searches were screened by title and abstract for relevance. Full-text articles that met inclusion criteria were reviewed. Data were extracted on:

- The type of musculoskeletal disorder
- Associated ocular findings
- Pathophysiological connections
- Diagnostic or surgical relevance for orthopaedics

No quantitative synthesis was performed, as this review aimed to provide a qualitative, integrative overview of literature findings. The results were organized around four core objectives:

- Identification of MSDs with ocular manifestations
- Pathophysiological mechanisms linking MSDs and ocular symptoms
- Clinical implications for orthopaedic specialists
- Current screening and management recommendations

### Overview of Musculoskeletal Disorders With Ocular Manifestations

Musculoskeletal disorders (MSDs) may present with ocular manifestations that reflect their systemic pathophysiology. For orthopaedic practitioners, recognizing these signs can aid in diagnosis, guide treatment planning, and ensure appropriate referral or interdisciplinary management. The table below summarizes key MSDs with their corresponding ocular findings and the mechanisms linking both systems (Table 1).

**Table 1:** Key Musculoskeletal Disorders With Characteristic Eye Findings

Musculoskeletal Disorder (MSD)	Ocular Finding	Pathophysiological Explanation
Rheumatoid arthritis	Dry eye, scleritis^1^	Autoimmune inflammation affects lacrimal glands and sclera
Marfan syndrome	Lens subluxation, retinal detachment^2^	FBN1 mutation weakens connective tissue in the eye
Osteogenesis imperfecta	Blue sclera^3^	Type I collagen defect causes scleral thinning, making underlying uveal tissue visible
Paget disease of bone	Optic neuropathy^4^	Skull remodeling can compress the optic nerve
Ankylosing spondylitis	Anterior uveitis^5^	HLA-B27–linked inflammation affects both joints and uveal tissue
Ehlers-Danlos syndrome	Double vision (strabismus), high myopia, retinal detachment, and blue sclera^7^	Collagen abnormalities lead to corneal thinning and laxity
Neurofibromatosis type 1	Lisch nodules^8^	NF1 gene mutation causes melanocytic iris hamartomas
Homocystinuria	Ectopia lentis, High myopia and retinal detachment^9^	Enzyme defect leads to homocysteine accumulation, weakening zonular fibers
Juvenile idiopathic arthritis	Chronic anterior uveitis^10^	Early-onset autoimmune disease targets both joints and eyes

FBN1, fibrillin-1; HLA-B27, human leukocyte antigen B27; NF1, neurofibromatosis type 1.

### Pathophysiological Mechanisms Linking MSDs and Ocular Symptoms

Rather than treating these findings as isolated phenomena, orthopaedic specialists can benefit from understanding how systemic abnormalities such as collagen defects, immune-mediated inflammation, or metabolic imbalance affect both the musculoskeletal and ocular systems. This knowledge enables better diagnostic interpretation, risk assessment, and collaboration with other specialties in managing complex patients.

1. Rheumatoid Arthritis (RA)

RA is a chronic autoimmune disease affecting synovial joints. Immune-mediated inflammation also targets the lacrimal glands and sclera, leading to dry eye disease and episcleritis/scleritis.^1^ These findings often reflect systemic disease activity and are relevant to orthopaedic surgeons managing joint replacement in patients on long-term immunosuppressants or corticosteroids.

2. Marfan Syndrome

This disorder results from mutations in the fibrillin-1 (FBN1) gene, impairing connective tissue integrity. Orthopaedically, it presents with scoliosis, joint laxity, and pectus deformities. Ocularly, lens subluxation (ectopia lentis) and high myopia are early clues because of zonular weakness.^2^ Recognizing these signs early can trigger cardiologic assessment and help mitigate perioperative cardiovascular risks associated with underlying aortic pathology.

3. Osteogenesis Imperfecta (OI)

OI is caused by mutations affecting type I collagen, leading to brittle bones and skeletal deformities. The same collagen defect weakens the sclera, resulting in the classic blue sclera appearance.^3^ From an orthopaedic viewpoint, this ocular sign may precede fractures and should prompt early evaluation for systemic bone fragility.

4. Paget Disease of Bone

Paget disease leads to abnormal bone remodeling, particularly in the skull and pelvis. When craniofacial bones are involved, optic nerve compression may occur.^4^ Although rare, these clinically significant eye symptoms can indicate advanced skull involvement, prompting imaging and influencing surgical risk assessment.

5. Ankylosing Spondylitis (AS)

AS is a seronegative spondyloarthropathy affecting the axial skeleton. It is strongly associated with HLA-B27, and up to 40% of patients develop acute anterior uveitis.^7^ These episodes may precede spinal symptoms and are a valuable diagnostic clue. Their recurrence also signals disease activity, which may affect surgical timing and the need for systemic anti-inflammatory treatment.

6. Ehlers-Danlos Syndrome (EDS)

EDS encompasses a group of connective tissue disorders that lead to joint hypermobility, frequent dislocations, and poor wound healing. Collagen abnormalities also cause lens instability and blue sclera.^8^ In orthopaedic care, these eye findings suggest systemic tissue fragility and inform surgical planning, especially in procedures involving soft tissues or spinal stabilization.

7. Neurofibromatosis Type 1 (NF1)

NF1 frequently presents with scoliosis, pseudarthrosis, and limb deformities. Its most common ocular manifestation is Lisch nodules, benign iris hamartomas.^9^ Although they do not impair vision or orthopaedic outcomes, they serve as an early diagnostic clue especially in pediatric patients with spinal abnormalities of unclear origin.

8. Homocystinuria

This metabolic disorder leads to elevated homocysteine, affecting connective tissue and increasing fracture risk. Lens dislocation is often the first sign.^10^ In orthopaedic patients with tall stature, long limbs, and scoliosis, this ocular finding may help differentiate homocystinuria from Marfan syndrome and guide medical management (e.g., vitamin B6 therapy).

9. Juvenile Idiopathic Arthritis (JIA)

JIA involves early-onset joint inflammation, particularly affecting knees, ankles, and wrists. In many cases, chronic anterior uveitis coexists and may precede joint symptoms.^11^ This is especially important for pediatric orthopaedic surgeons, as silent ocular inflammation can lead to vision loss without regular screening, even in children undergoing joint evaluations.

### Clinical Implications for Orthopaedic Specialists

In orthopaedic practice, ocular manifestations are often overlooked during routine assessment of musculoskeletal disorders. However, these signs can carry significant clinical value. Rather than serving purely diagnostic roles, ocular findings can influence surgical timing, risk stratification, perioperative management, and guide multidisciplinary referral. For orthopaedic surgeons, especially those treating pediatric, syndromic, or inflammatory cases awareness of relevant ocular signs may enhance the accuracy, safety, and efficiency of care.

In this context, ocular assessment does not imply a formal ophthalmologic examination. Rather, it refers to basic visual inspection and recognition of overt ocular abnormalities during routine orthopaedic evaluation, with referral to ophthalmology when indicated.

1. Early Recognition and Referral Value

Orthopaedic teams are frequently the first specialists to evaluate patients with skeletal deformities or unexplained fractures. Ocular signs such as blue sclera, lens subluxation, or uveitis can serve as early indicators of systemic disease.^12^ Their presence should prompt targeted referral to genetics, rheumatology, or ophthalmology, allowing for more accurate diagnosis and avoidance of isolated orthopaedic treatment in complex syndromic cases.

For example, identifying a subtle ocular finding during a preoperative examination in a patient with hypermobility or scoliosis could signal an undiagnosed systemic disorder like Marfan or Ehlers-Danlos syndrome.^2,8,13^ Early detection in such cases enables proactive cardiac screening, imaging, or medical management before surgical intervention.

2. Impact on Surgical Planning and Risk Management

Certain ocular manifestations are markers of systemic disease activity (e.g., scleritis in RA, active uveitis in AS or JIA).^1,5,11^ These signs may indicate heightened systemic inflammation and increase the risk of delayed healing or infection.^12^ Their detection should prompt coordination with the managing rheumatologist and, when necessary, delay of elective orthopaedic procedures until disease control is achieved.^14^

In hereditary connective tissue disorders, ocular features suggestive of collagen fragility (e.g., keratoconus or lens instability) should raise red flags for soft-tissue vulnerability. These features may be associated with poor wound healing and increased intraoperative bleeding, factors that directly influence implant selection and surgical approach.^15^

3. Monitoring and Multidisciplinary Collaboration

In pediatric orthopaedic patients, routine ophthalmologic evaluation is not typically part of orthopaedic assessment; however, awareness of high-risk conditions such as Juvenile Idiopathic Arthritis remains essential, as uveitis may be asymptomatic and progress silently.^11,16^ Lack of awareness can lead to delayed ophthalmology referral and irreversible vision damage, even while orthopaedic care proceeds.

Collaborative care pathways where ophthalmologists and orthopaedic surgeons maintain mutual awareness of systemic disease manifestations can lead to earlier interventions, more accurate prognosis, and avoidance of systemic complications during orthopaedic treatment courses.^17^

4. Avoiding Misdiagnosis and Unnecessary Investigations

In cases of unexplained musculoskeletal symptoms, the presence of characteristic eye signs (e.g., Lisch nodules in NF1 or lens dislocation in homocystinuria) can prevent misdiagnosis or excessive imaging.^9,10^ Recognizing these subtle ocular clues during orthopaedic assessment can narrow the differential diagnosis and help prioritize necessary investigations saving time, resources, and patient distress.

Given the subtle nature of many ocular manifestations, targeted educational measures may improve orthopaedic surgeons' ability to recognize clinically relevant findings. These may include brief training modules, interdisciplinary case discussions with ophthalmology, and inclusion of basic ocular inspection principles in orthopaedic residency curricula.

### Current Screening and Management Guidelines

Despite growing recognition of ocular involvement in musculoskeletal disorders (MSDs), standardized screening protocols remain limited in orthopaedic practice. This gap may delay diagnosis of systemic disease, increase surgical risks, or result in missed opportunities for early intervention. Integrating ocular screening into musculoskeletal care especially for high-risk patient groups can improve diagnostic accuracy, treatment planning, and overall outcomes.

In this section, screening refers to identifying patients who require ophthalmology referral based on clinical risk factors and apparent ocular findings, rather than performing a formal eye examination within orthopaedic practice.

**Figure 1:**
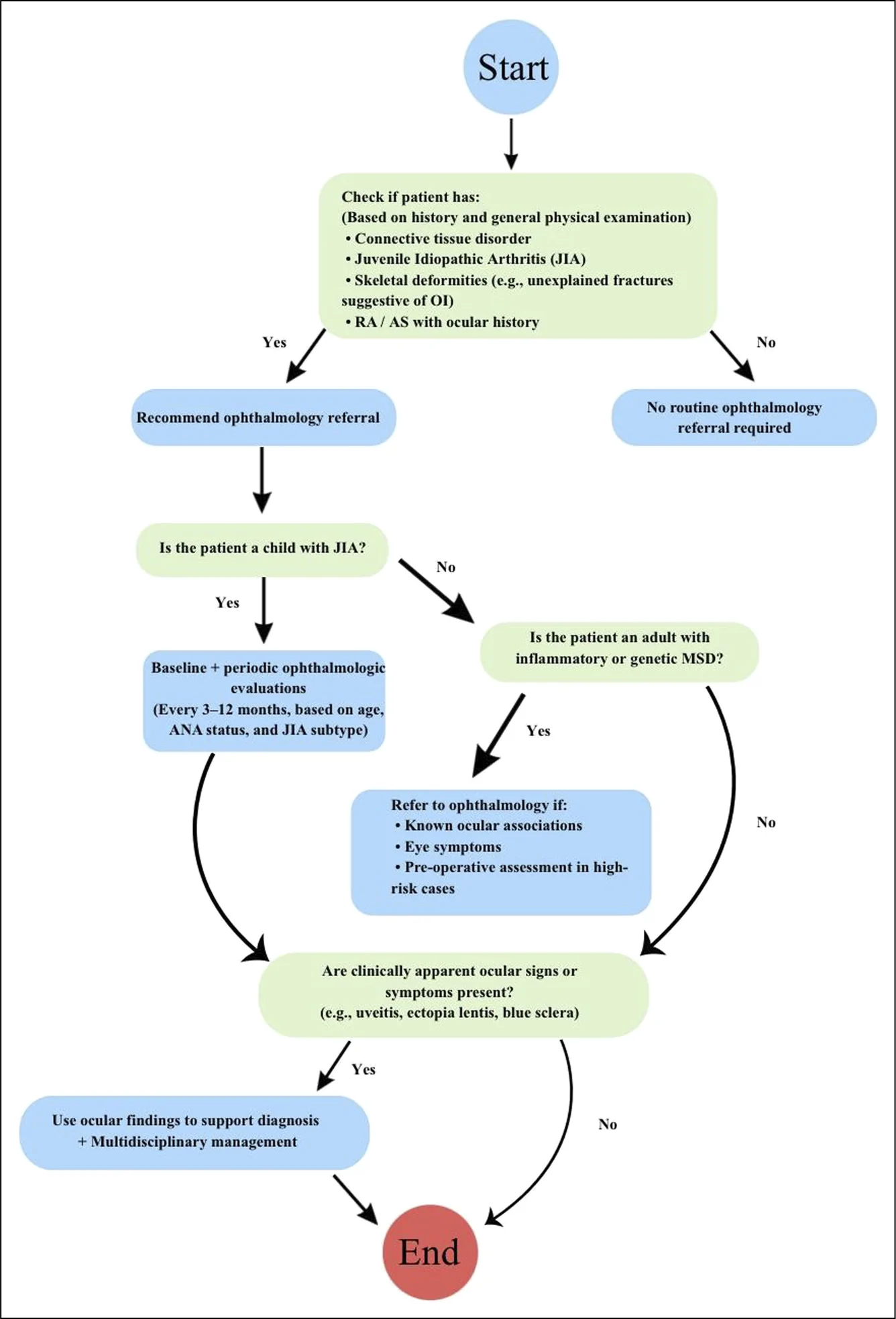
Diagram demonstrating algorithm for ophthalmology referral and ocular awareness in orthopaedic patients with musculoskeletal disorders.

1. Who Should Be Referred for Ophthalmologic Evaluation (Figure 1)?

Routine ophthalmologic evaluation is not needed for all orthopaedic patients, but it is highly recommended for individuals with:

- Confirmed or suspected connective tissue disorders (e.g., Marfan syndrome, Ehlers-Danlos syndrome)
- Juvenile idiopathic arthritis (JIA) or other pediatric inflammatory arthritides
- Unexplained fractures or skeletal deformities suggestive of osteogenesis imperfecta^18^
- Chronic inflammatory arthropathies like rheumatoid arthritis or ankylosing spondylitis, especially with ocular history

In these cases, ocular findings such as ectopia lentis, blue sclera, or asymptomatic uveitis can support diagnosis and guide multidisciplinary management.^2,3,17^

2. Timing and Frequency of Eye Evaluations

Pediatric patients with JIA require baseline and periodic eye exams, even in the absence of symptoms. Uveitis may be asymptomatic but progressive, leading to permanent vision loss without early detection.^11,19^ Screening intervals typically range from every 3 to 12 months depending on age, antinuclear antibodies (ANA) status, and JIA subtype.^20^

Adult patients with inflammatory or genetic MSDs should be referred to ophthalmology:

- At diagnosis, if the disease has known ocular associations
- When eye symptoms (e.g., redness, pain, vision changes) emerge
- surgery in syndromic or high-risk cases to assess for corticosteroid complications or tissue fragility^21^

3. Interdisciplinary Management Recommendations

Effective care requires close collaboration between orthopaedic specialists, ophthalmologists, and rheumatologists or geneticists. Ocular signs should be flagged as indicators of systemic disease activity.^12^ Coordination is especially important:

- When adjusting systemic immunosuppressants before or after surgery^22^
- In syndromic patients undergoing corrective procedures (e.g., scoliosis repair, joint reconstruction)
- When ocular findings confirm or suggest a multisystem diagnosis (e.g., Lisch nodules in NF1)

4. Institutional and Guideline Gaps

Most orthopaedic clinical practice guidelines currently lack integrated ocular screening protocols for high-risk MSD populations. This represents a missed opportunity to prevent visual complications, enhance diagnosis, and ensure comprehensive care.

Suggested practice improvements include the following:

- Adding ocular referral prompts to preoperative orthopaedic checklists.
- Training orthopaedic residents to recognize basic eye signs linked to MSDs.
- Embedding ophthalmology screening recommendations into shared care pathways with pediatrics, rheumatology, and genetics.

## Conclusion

Ocular manifestations are a clinically relevant but often underrecognized component of musculoskeletal disorders (MSDs). In many cases, these eye findings can serve as early indicators of systemic disease, markers of ongoing inflammatory activity, or red flags for surgical risk. For orthopaedic specialists, such manifestations are not incidental; they can inform diagnosis, guide perioperative planning, and prompt appropriate multidisciplinary referrals.

This narrative review highlighted a range of MSDs where ocular involvement plays a diagnostic or prognostic role, particularly in genetic, connective tissue, and inflammatory conditions. Although some signs, like blue sclera or lens subluxation, are syndromic clues, others such as uveitis may signal active disease requiring medical stabilization before orthopaedic intervention.

Despite the growing body of evidence, systematic identification of patients requiring ophthalmology referral remains inconsistently applied in orthopaedic protocols. Greater awareness and targeted referral of high-risk patients especially pediatric, syndromic, or autoimmune cases can improve diagnostic efficiency, reduce complications, and support holistic patient care.

Targeted educational initiatives and interdisciplinary collaboration may further enhance orthopaedic clinicians' ability to recognize clinically relevant ocular findings and act upon them appropriately. Integrating ocular findings into musculoskeletal assessment represents a practical and impactful step toward more comprehensive orthopaedic management.
